# Altered functional activity of the precuneus and superior temporal gyrus in patients with residual dizziness caused by benign paroxysmal positional vertigo

**DOI:** 10.3389/fnins.2023.1221579

**Published:** 2023-10-12

**Authors:** Cunxin Lin, Dan Liu, Yueji Liu, Zhengwei Chen, Xiue Wei, Haiyan Liu, Kai Wang, Tengfei Liu, Lijie Xiao, Liangqun Rong

**Affiliations:** ^1^Department of Neurology, The Second Affiliated Hospital of Xuzhou Medical University, Xuzhou, Jiangsu, China; ^2^Graduate School of Xuzhou Medical University, Xuzhou, Jiangsu, China

**Keywords:** amplitude of low frequency fluctuation, benign paroxysmal positional vertigo, residual dizziness, resting state function magnetic resonance imaging, functional connectivity

## Abstract

**Objective:**

Benign paroxysmal positional vertigo (BPPV) is a common clinical vertigo disease, and the most effective treatment for this disease is canal repositioning procedures (CRP). Most patients return to normal after a single treatment. However, some patients still experience residual dizziness (RD) after treatment, and this disease’s pathogenesis is currently unclear. The purpose of this study is to explore whether there are abnormal brain functional activities in patients with RD by using resting-state functional magnetic resonance imaging (rs-fMRI) and to provide imaging evidence for the study of the pathogenesis of RD.

**Materials and methods:**

The BPPV patients in the Second Affiliated Hospital of Xuzhou Medical University had been included from December 2021 to November 2022. All patients had been received the collection of demographic and clinical characteristics (age, gender, involved semicircular canal, affected side, CRP times, BPPV course, duration of RD symptoms, and whether they had hypertension, diabetes, coronary heart disease.), scale assessment, including Dizziness Handicap Inventory (DHI), Hamilton Anxiety Inventory (HAMA), Hamilton Depression Inventory (HAMD), rs-fMRI data collection, CRP treatment, and then a one-month follow-up. According to the follow-up results, 18 patients with RD were included. At the same time, we selected 19 healthy individuals from our hospital’s physical examination center who matched their age, gender as health controls (HC). First, the amplitude of low-frequency fluctuations (ALFF) analysis method was used to compare the local functional activities of the two groups of subjects. Then, the brain regions with different ALFF results were extracted as seed points. Functional connectivity (FC) analysis method based on seed points was used to explore the whole brain FC of patients with RD. Finally, a correlation analysis between clinical features and rs-fMRI data was performed.

**Results:**

Compared to the HC, patients with RD showed lower ALFF value in the right precuneus and higher ALFF value in the right superior temporal gyrus (STG). When using the right STG as a seed point, it was found that the FC between the right STG, the right supramarginal gyrus (SMG), and the left precuneus was decreased in RD patients. However, no significant abnormalities in the FC were observed when using the right precuneus as a seed point.

**Conclusion:**

In patients with RD, the local functional activity of the right precuneus is weakened, and the local functional activity of the right STG is enhanced. Furthermore, the FC between the right STG, the right SMG, and the left precuneus is weakened. These changes may explain the symptoms of dizziness, floating sensation, walking instability, neck tightness, and other symptoms in patients with RD to a certain extent.

## 1. Introduction

Benign paroxysmal positional vertigo (BPPV) is the most common peripheral vertigo, accounting for about 40% (Kim et al., 2021). According to statistics, the lifetime prevalence rate for women is 3.2%, and for men it is 1.6%. The overall prevalence rate is 2.4% for the adult population and increases with age (von Brevern et al., 2007; Nuti et al., 2020). The disease is characterized by transient vertigo attack and specific nystagmus with the change of body position (getting up, turning over, turning head). Each attack lasts for several seconds, and the patient often complains of a sense of rotation, accompanied by nausea and vomiting in severe cases (von Brevern et al., 2015; Imai and Inohara, 2022). Currently, the canal repositioning procedures (CRP) is the most effective method to treat BPPV, and most patients can recover to normal after one time (Fernández et al., 2015; Cole and Honaker, 2022). However, clinical studies have found that 31–60% of patients still have symptoms such as dizziness, floating, unstable walking, neck tightness, and other symptoms after receiving treatment. However, there is no vertigo and specific nystagmus, and the position-induced test results are negative. These clinical symptoms are residual dizziness (RD; Martellucci et al., 2016; Giommetti et al., 2017; Ke et al., 2022). Cristina Vaduva et al. found that 47.2% of RD patients had balance disorders, and older people over 65 years were more likely to have residual dizziness (Vaduva et al., 2018). The RD symptoms seriously affected patients’ daily work and life, leading to accidental falls in some elderly patients and increasing the medical economic burden. At the same time, it could also cause anxiety and depression in some patients (Martellucci et al., 2016), and even developed into persistent postural perceptual dizziness (PPPD; Staab et al., 2017; Makarov et al., 2020). Although we have recognized the harm of this disease, the pathogenesis of residual dizziness is not particularly clear at present, and more clinical studies are urgently needed to explore.

Faralli et al. suggested that the generation of RD may be related to the compensatory mechanism of the vestibular center (Faralli et al., 2016; Makarov et al., 2020). Mario Faralli et al. found that patients with earlier visits and treatment were less likely to develop RD symptoms. They believed that the loss of otolith would cause functional asymmetry of the bilateral peripheral vestibular function, triggering the compensatory mechanism of the vestibular center. Although CRP treatment, the compensatory mechanism of the brain was relatively slow and took some time to complete, and it could not quickly adapt to the changes in the peripheral vestibular function state, thus causing the production of RD symptoms (Faralli et al., 2016). However, there is not enough evidence for this view at present.

In recent years, resting-state functional magnetic resonance (rs-fMRI), a non-invasive technology, has been applied in clinical research. It used the imaging mechanism dependent on blood oxygen level to reflect the functional activity of the brain, which can helped us to study whether the functional activities of the brain in patients were abnormal (Logothetis, 2008; Glover, 2011). The technology has been widely used in the scientific research of Alzheimer’s disease (AD; Chandra et al., 2019), cognitive impairment (CI; Talwar et al., 2021), Parkinson’s disease (PD; Boutet et al., 2021). Using rs-fMRI, Zhu et al. found that cerebellar regional homogeneity (Re Ho) values, degree centrality (DC) values, and brain stem fractional amplitude of low-frequency fluctuations (fALFF) values were significantly increased in patients with BPPV, suggesting that the local brain region produced functional compensation in order to adapt to the state after otolith restoration. At the same time, the study used high-resolution structural image analysis to find a decrease in cerebellar gray matter volume in BPPV patients, which they believed may be reversed to eliminate vertigo and nystagmus (Zhu et al., 2021). Fu et al. also used rs-fMRI to find that the amplitude of low-frequency fluctuations (ALFF) value of bilateral precuneus in patients with RD was significantly lower than in patients without RD symptoms. It may lead to the decreased processing ability of vestibular information and the inability to complete the integration of vestibular and visual-related information (Fu et al., 2022).

Although there have been studies related to rs-fMRI of RD, the previous studies were limited to exploring the changes in local brain functional activities, which could only reflect the strength of functional activity in a particular brain area. They could not reflect functional connectivity (FC) changes between brain networks. In addition, due to the different populations selected in the two studies, it was impossible to compare the two studies, and the two studies were single-center studies, which was not enough to represent the whole group, and more studies are needed to verify in the further. The ALFF reflected the spontaneous neural activity of local brain regions by detecting the intensity of spontaneous activity of neurons in a specific frequency range (Gong et al., 2020; Sun et al., 2020). The seed-based FC calculated the time series correlation between the average time series of the region of interest (ROI) and other voxels in the whole brain to reflect the integration function of the brain networks (Baggio et al., 2015). By combining the ALFF and FC methods, the changes in brain functional activity in RD patients can be comprehensively discussed from the perspectives of local function and global networks. Therefore, based on previous studies, this study uses the ALFF analysis method and seed-based FC method to explore whether there are abnormalities in the brain functional activities of patients with RD in order to provide a more theoretical basis for the study of the pathogenesis of RD from the perspective of imaging.

## 2. Materials and methods

### 2.1. Study subject

Fifty-three BPPV patients who sought medical treatment at the outpatient clinics or hospital wards of the Second Affiliated Hospital of Xuzhou Medical University from December 2021 to November 2022, all of whom were right-handed and successfully received CRP, had been included. According to later follow-up results, 18 patients with RD were eventually included. At the same time, we selected 19 healthy individuals from our hospital’s physical examination center who matched their age, gender as HC. Furthermore, the diagnostic criteria for BPPV were based on the criteria established by the Barany Society in 2015 (von Brevern et al., 2015). The following patients were excluded: (1) patients with multiple canal-type BPPV and superior canal-type BPPV; (2) patients with other vertigo disorders. For example, vestibular migraine (VM), persistent postural-perceptual dizziness (PPPD), Meniere’s disease (MD), vestibular neuritis (VN), malignant vertigo (MV); (3) contraindications to MRI examination; (4) CRP failed; (5) unable to cooperate with follow-up; (6) patients who had already developed RD symptoms before CRP.

The Second Affiliated Hospital Ethics Committee of Xuzhou Medical University approved this study, and all subjects had signed the informed consent and had volunteered to participate.

### 2.2. Demographic and clinical characteristics

Demographic information had been recorded for all enrolled BPPV patients at baseline, including age, gender, and patient clinical characteristics (including involved semicircular canal, affected side, CRP times, BPPV course, and whether they had hypertension, diabetes, coronary heart disease) and rs-fMRI data had been collected. The duration of RD symptoms in the RD group was recorded according to the follow-up results for 1 month. In addition, all patients enrolled with BPPV had been assessed with relevant scales at baseline, including the Dizziness Handicap Inventory (DHI), Hamilton Anxiety Inventory (HAMA), and Hamilton Depression Inventory (HAMD).

### 2.3. Canalith repositioning procedure

The Dix-Hallpike test is a positioning nystagmus test used to diagnose posterior-canal-type BPPV. The Roll test is a positional nystagmus test used to diagnose lateral-canal-type BPPV. The CRP for posterior-canal-type BPPV is called the Epley maneuver, and the CRP for lateral-canal-type BPPV is called the Gufoni or Barbecue maneuver. All patients completed the first CRP and underwent the Dix-Hallpike test and Roll test 30 min later. The CRP treatment was successful if the two test were negative and the patient did not complain of vertigo. If the two test were positive, repeat CPR treatment up to two times. Otherwise, the CRP treatment was considered a failure.

### 2.4. Follow up

BPPV patients who met the inclusion and exclusion criteria were recorded and followed up by outpatient follow-up or telephone inquiries. Patients with BPPV were followed up 24 hours after CRP and then weekly for up to 1 month. Structured questions were used to determine whether patients were RD patients: (1) After CRP, did they still feel like they were spinning around when they turned their heads or got up? If the patient answered “yes”, it was considered a recurrence of BPPV, and the patient was advised to seek further treatment at the clinic. If the patient answered “no”, the patient was asked the following question. (2) Did the patient experience dizziness, discomfort, unstable walking, floating sensation, and neck tightness after CRP? If the patient answered “yes”, the patient was considered to have RD symptoms. The duration of RD symptoms was then recorded at a later follow-up.

### 2.5. Magnetic resonance data acquisition

A 3.0 T magnetic resonance scanner (GE DISCOVERY 3.0 T magnetic resonance instrument with the 8-channel cranial coil, United States) was used to collect images. All subjects were asked to wear clothes that did not contain metal, close their eyes, keep their bodies stationary, keep their entire bodies relaxed, and avoid falling asleep during the scanning process. In addition, soundproof earplugs were worn during the scanning process to avoid the interference of MRI machines on patients. A conventional cranial MRI scan was performed to check for organic lesions. Blood oxygen levels dependent (BOLD) images were collected using an echo planar imaging (EPI) sequence, repetition time (TR) = 2000 ms, echo time (TE) = 30 ms, flip angle (FA) = 90°, field of view (FOV) = 200 × 200 mm, number of slice = 38, acquisition matrix = 64 × 64, voxel size = 3.0× 3.0 × 3.0 mm^3^, slice thickness = 3 mm, gap = 0 mm, 210 contiguous functional volumes were acquired. The T1-weighted anatomic images were collected using a 3D-BRAVO sequence: TR = 2,500 ms, TE = 3.5 ms, FA = 8°, acquisition matrix = 256 × 256, slice thickness = 1 mm, number of slices = 156.

### 2.6. rs-fMRI data processing and analysis

#### 2.6.1. rs-fMRI data pre-processing

The image data were processed using DPARSFA7.0^1^ software based on the MATLAB2022a platform. (1) data format conversion: convert image data from DICOM to NIFTI format. (2) remove the first ten time points: the magnetic resonance machine signal may be unstable during the initial scan, so removing the first ten time points could ensure the stability of the data. (3) slice timing correction: perform time level correction to ensure all brain voxels were obtained simultaneously. The total number of slices scanned in this study was 38, and the ascending scans were carried out from the even slice, and the first slice was selected as the reference slice. (4) head motion correction: correct the slight head movement of the subject at various time points during the scanning process to ensure the accuracy of position information. Subjects whose heads shifted more than 3.0 mm in X, Y, or Z directions or rotated more than 3° would be excluded. (5) regression covariates: white matter signals, cerebrospinal fluid signals, and frame-wise displacement parameters of Friston 24 were used as confounding covariates for regression. (6) spatial standardization: in order to solve the problem of brain morphological differences among different subjects and inconsistent spatial positions during scanning, the DARTEL registration method was used to register the structural space and functional space to realize the conversion from a single space to the standard Montreal Neurological Institute (MNI) space. Furthermore, resampling at a resolution of 3 mm × 3 mm × 3 mm. (7) spatial smoothing: the Gaussian kernel of 4 mm × 4 mm × 4 mm was used for smoothing, reducing registration errors and improving the normality of the data.

#### 2.6.2. ALFF analysis

The power spectrum was obtained by converting the time series of each voxel into frequency domain data using the fast Fourier transform algorithm. The average square root of the power spectrum in the range of 0.01 to 0.08 Hz was calculated as the ALFF value. Then, the ALFF value of each subject was converted into a z-score for voxel-by-voxel inter-group comparison.

Age, gender, and head motion parameters (Mean FD_Jenkinson) were covariates. The two sample *t*-test was used to compare the ALFF values in each voxel of the two groups. Multiple comparisons using the Threshold Free Cluster Enhancement (TFCE) permutation test can easily control the false positive rate and reproducibility existing in statistics and 5,000 permutation times and the prominent cluster size >40 voxels were performed in this work (two-tailed, threshold level: *p* < 0.05; Eklund et al., 2016; Chen et al., 2018).

#### 2.6.3. Seed-based FC analysis

Based on ALFF, we took the brain regions with significant differences in ALFF values between the two groups as seed points and used the seed-to-voxel method to calculate the FC between ROI and other voxels in the whole brain. We choose the right precuneus and the right superior temporal gyrus (STG) as ROIs. The average time series for each ROI was extracted from the pre-processed images. Then, Pearson correlation coefficients between ROI and other brain voxels were calculated and converted to z-scores using *Fisher* r-to-z transformation.

Similarly, we used the two sample *t* test to analyze the FC differences between the two ROIs and other brain regions of the two groups of subjects. Age, gender, and head motion parameters (Mean FD_Jenkinson) were covariates. We used the TFCE permutation test for multiple comparison correction and 5,000 permutation times, and the prominent cluster size >40 voxels were performed in this work (two-tailed, threshold level: *p* < 0.05; Eklund et al., 2016; Chen et al., 2018).

#### 2.6.4. Correlation analysis

Age and gender were used as covariates, the different brain regions of ALFF and FC in each RD patient were extracted, z-values were calculated, and Pearson partial correlation analysis were performed with the clinical characteristics of patients. When *p* < 0.05, it indicated a statistically significant difference.

### 2.7. Statistical analysis of data

The clinical characteristic data of all subjects were analyzed using SPSS 25.0 software (SPSS Institute Inc., Chicago, IL, United States). Firstly, the *Shapiro–Wilk* test was used to test the normality of measurement data in the clinical characteristics of the two groups of subjects. When *p* > 0.05, the data was considered to conform to normal distribution. For the measurement data conforming to the normal distribution, the mean ± standard deviation was used for statistical description, and the two sample *t* test was conducted for statistical analysis. When *p* < 0.05, the differences were considered statistically significant. For the measurement data that did not conform to the normal distribution, the median (*inter-quartile spacing*) was used for statistical description, and the non-parametric rank sum test was used for statistical analysis. The test type was *Mann–Whitney/Wilcoxon*, and the differences were considered statistically significant when *p* < 0.05. For the counting data, the rate was used for statistical description, and the *Chi-square* test was used for statistical analysis. When *p* < 0.05, the differences were considered statistically significant.

## 3. Results

### 3.1. Comparison of general clinical characteristics between RD group and HC group

According to the inclusion and exclusion criteria, as well as the later follow-up results, we included a total of 18 patients with RD, whose average age was 59.17 ± 7.06 years [mean ± standard deviation], including 12 females and 6 males, with female–male ratio of 12/6. At the same time, 19 healthy adults were collected from the physical examination center as HC, with an average age of 61.85 ± 6.39 years [mean ± standard deviation], including 10 females and 9 males, with a ratio of 10/9 females to males.

Statistical analysis showed no significant differences in age and gender between the two groups (*p* > 0.05, Table 1).

**Table 1:** Clinical characteristics between the RD and HC group.

	RD (*n* = 18)	HC (*n* = 19)	Value of *p* (*t/χ*^2^)
**Gender**			
Female	12 (66.7%)	10(53%)	0.508^a^
Male	6 (33.3%)	9(47%)	
**Age (years)**	59.17 ± 7.06	61.85 ± 6.39	0.520^b^
**Involved canal (%)**		-	-
Posterior	7 (38.9%)		
Horizontal	11 (61.1%)		
**Affected side (%)**		-	-
Right	11 (61.1%)		
Left	7 (38.9%)		
**RD duration (days)**	8.26 ± 7.37	-	-
**Duration of BPPV (days)**	17 (7.5)	-	-
**CRP times**	5.50 (3.00)	-	-
**DHI scores**	28 (8)	-	-
**DHI-P**	10 (4.5)	-	-
**DHI-E**	10(6)	-	-
**DHI-F**	7(4)	-	-
**HAMA scores**	16.11 ± 5.42	-	-
**HAMD scores**	6.50 (2.25)	-	-
**Hypertension (%)**		-	-
Yes	9 (50%)		
No	9 (50%)		
**Diabetes (%)**		-	-
Yes	5 (27.80%)		
No	13 (72.20%)		
**Coronary heart disease (%)**		-	-
Yes	8 (44.4%)		
No	10 (55.6%)		

RD, residual dizziness; HC, healthy control; DHI, dizziness handicap inventory; DHI-P, dizziness handicap inventory-physical subscale; DHI-E, dizziness handicap inventory-emotional subscale; DHI-F, dizziness handicap inventory-functional subscale; HAMA, hamilton anxiety inventory; HAMD, Hamilton depression inventory; ^a^Chi-square test; ^b^Two sample *t*-test.

### 3.2. Comparison of ALFF results between RD group and HC group

**Figure 1:**
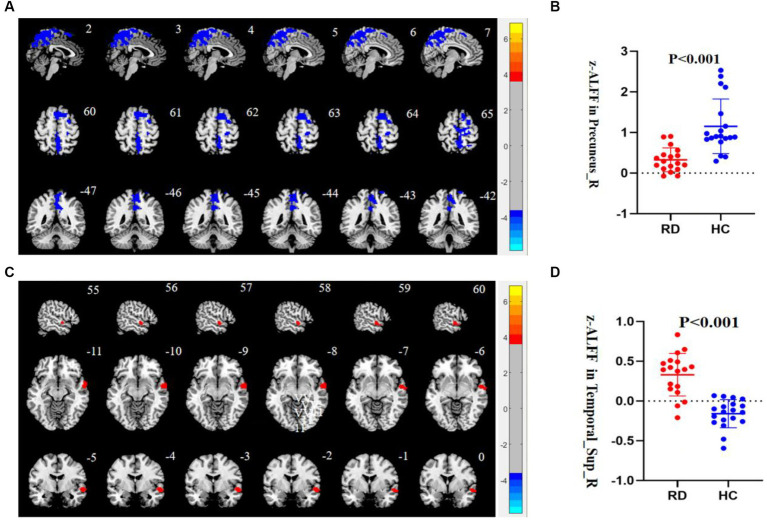
**(A)** Compared to HC, the ALFF value of the right precuneus is decreased in patients with RD (*p* < 0.05, TFCE permutation test corrected). Blue represents a significant decrease in ALFF value. **(B)** The z-ALFF value of the right precuneus between the RD group and HC group is statistically different (*p* < 0.001, uncorrected). The red scatter plot represents the z-ALFF value in the right precuneus of each participant in the RD group, and the blue scatter plot represents the z-ALFF value in the right precuneus of each participant in the HC group. **(C)** Compared to HC, the ALFF value of the right STG is increased in patients with RD (*p* < 0.05, TFCE permutation test corrected). Red represents a significant increase in ALFF value. **(D)** The z-ALFF value of the right STG between the RD and HC group is statistically different (*p* < 0.001, uncorrected). The red dot represents the z-ALFF value in the right STG of each participant in the RD group, and the blue dot represents the z-ALFF value in the right STG of each participant in the HC group.

It was shown that the RD group exhibited lower ALFF values in the right precuneus than the HC group. On the other hand, the RD group exhibited higher ALFF values in the right STG than HC group (*p* < 0.05, TFCE permutation test corrected, see Table 2; Figure 1).

**Table 2:** The brain regions show significant differences in ALFF values between the RD and HC group.

Brain region (AAL/Brodmann)	Number of voxels	MNI coordinates (x, y, z)	Peak *t* value
Precuneus_R/BA5_R	1,261	3, −45, 63	−5.88
Temporal_Sup_R/BA22_R	58	57, −3, −9	6.89

MNI, Montreal Neurologic Institute; R, right; AAL, anatomical automatic labeling; BA, Brodmann area; Precuneus_R/BA5_R, the right precuneus; Temporal_Sup_R/BA22_R, the right STG.

### 3.3. Comparison of FC results between RD group and HC group

**Figure 2:**
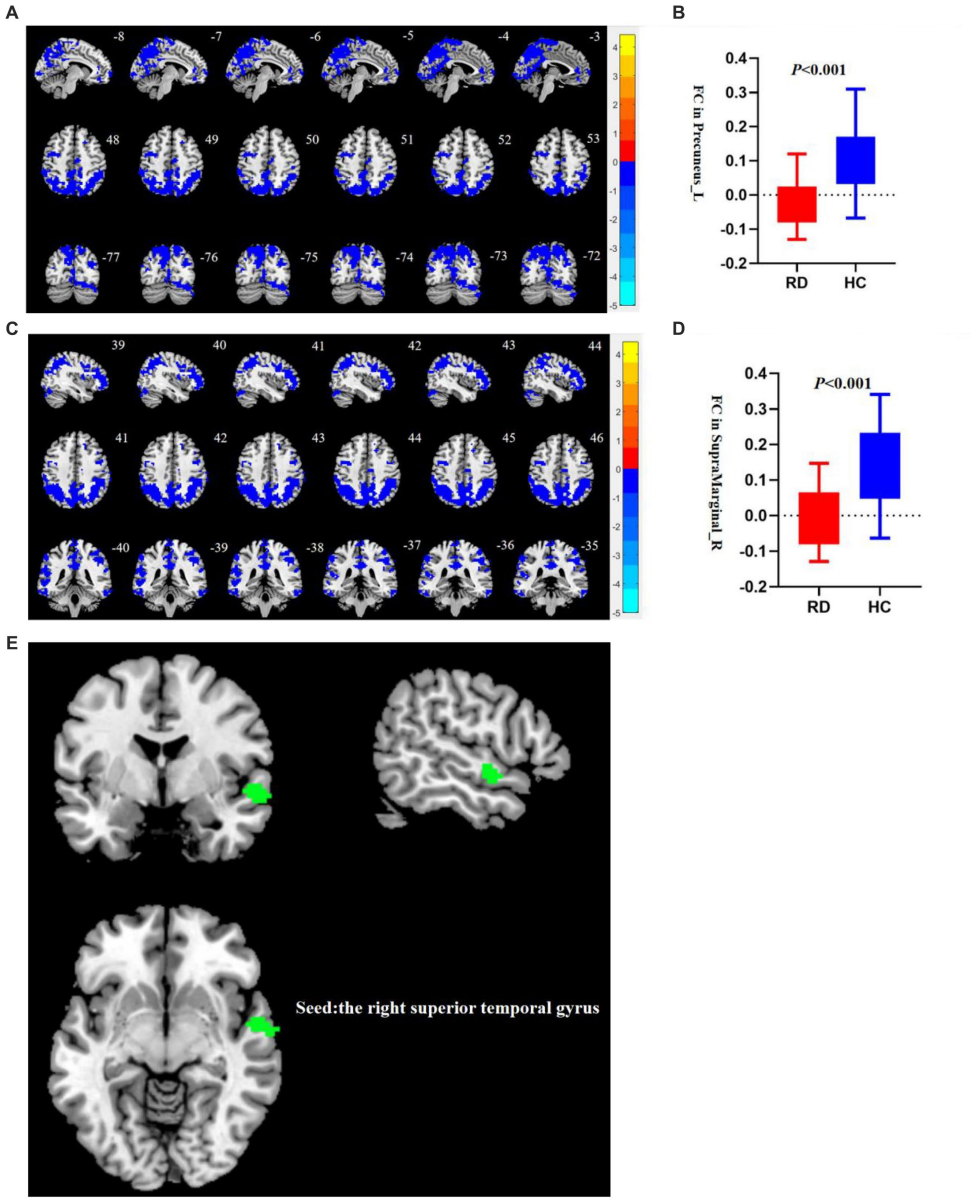
**(A)** Compared to HC, the FC between the right STG and the left precuneus decreases in patients with RD (*p* < 0.05, TFCE permutation test corrected). Blue represents a significant decrease in functional connectivity. **(B)** There are statistical differences in FC values between the right STG and the left precuneus between the RD and HC groups (*p* < 0.001, uncorrected). The red box plot represents the z-FC value between the right STG and the left precuneus in the RD group, and the blue box plot represents the z-FC value between the right STG and the left precuneus in the RD group. **(C)** Compared to HC, the FC between the right STG and the right SMG decreases in patients with RD (*p* < 0.05, TFCE permutation test corrected). Blue represents a significant decrease in FC. **(D)** There are statistical differences in FC values between the right STG and the right SMG between the RD and HC groups (*p* < 0.001, uncorrected). The red box plot represents the z-FC value between the right STG and the right SMG in the RD group, and the blue box plot represents the z-FC value between the right STG and the right SMG in the RD group. **(E)** The right STG is used as the seed point. Green represents the right STG.

Using the right STG as a seed point, the RD group showed decreased FC between the right STG and the left precuneus, as well as decreased FC between the right STG and the right supramarginal gyrus (SMG; *p* < 0.05, TFCE permutation test corrected, see Table 3; Figure 2). No abnormalities were observed in the FC when using the right precuneus as a seed point.

**Table 3:** With the right STG as the seed point, the brain regions show significant FC differences between the RD and HC group.

ROI	Brain region (AAL/Brodmann)	Number of voxels	MNI coordinates (x, y, z)	Peak *t* value
Temporal_Sup_R	Precuneus_L/BA7_L	504	−9, −72, 51	−5.02
	SupraMarginal_R/BA40_R	213	42, −39, 42	−5.00

ROI, region of Interest; L, left; Precuneus_L/BA7_L, the left precuneus; SupraMarginal_R/BA40_R, the right STM.

### 3.4. Correlation analysis

**Figure 3:**
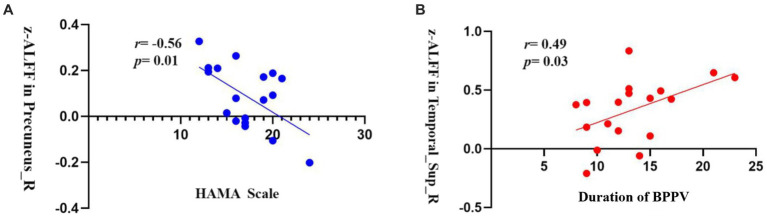
**(A)** The z-ALFF value of the right precuneus is negatively correlated with the HAMA anxiety scale in the RD group (*r* = −0.56, *p* = 0.01, uncorrected); **(B)** The z-ALFF value of the right STG is positively correlated with the duration of BPPV in the RD group (*r* = 0.49, *p* = 0.03, uncorrected).

In patients with RD, there was a significant negative correlation between the z-ALFF value in the right precuneus and the HAMA anxiety scale (*r* = −0.56, *p* = 0.01, uncorrected, Figure 3). Additionally, there was a significant positive correlation between the z-ALFF value in the right STG and the duration of BPPV (*r* = 0.49, *p* = 0.03, uncorrected, Figure 3).

## 4. Discussion

### 4.1. The ALFF value of the right precuneus in RD patients decreases

At present, rs-fMRI is one of the most commonly used methods to evaluate brain functional activities, and the exploration of brain functional activities mainly includes local functional activities and whole-brain functional connections. In our study, we first used the ALFF method to analyze the brain’s local functional activities of patients with RD. The ALFF value represents the strength of the functional activity of a particular brain area, and the higher the ALFF value, the stronger the functional activity of the brain area (Yang et al., 2020; Roffet et al., 2022). Our study found decreased ALFF values in the right precuneus in RD patients, indicating reduced functional activity in this region compared to HC. Previous studies have found that the precuneus is involved in many high-level cognitive functions, such as memory and information processing related to self-cognition (Thomas et al., 2019; Zhao et al., 2020), motor perception and emotion regulation (Fransson and Marrelec, 2008; Kovács et al., 2008). Wataru Sato, a psychologist at Jingdong University in Japan, analyzed magnetic resonance data from healthy people and found that people with higher happiness and life satisfaction had larger gray matter volume in the precuneus. This study suggested that the precuneus may have a function for processing aspects of human psychology (Sato et al., 2015). Our study discovered that the functional activity of the right precuneus in RD patients was weakened. It may be because patients with RD are exposed to vertigo for a long time and cannot carry out routine work and life, resulting in some psychological issues (such as anxiety and depression). In addition, through the clinical scale evaluation of RD patients, we also found that RD patients had certain anxiety and depression. BPPV patients had a sense of spinning, and there were specific nystagmus, severe nausea, and vomiting symptoms called vertigo. However, the symptoms of RD patients were discomfort, unsteady walking, lack of specific nystagmus, and no motor illusion, which was called dizziness (Brandt et al., 2014; Johkura, 2021). Previous studies have shown that dizziness is often associated with anxiety, depression, and panic disorder (Karatas, 2008). A systematic review and meta-analysis also suggested that RD may be a syndrome caused by a combination of multiple factors that can be explained from a psychological/physiological perspective (Ke et al., 2022). The correlation analysis in this study also found that the higher the score on the HAMA anxiety scale, the lower the ALFF value of RD patients, which indicates that the more serious the anxiety, the weaker the brain functional activity of the precuneus. Wang et al. found that small-dose betahistine plus cognitive behavioral therapy after CRP in BPPV patients was better than large-dose betahistine in relieving depression and anxiety symptoms and significantly improved dizziness and balance disorders in RD patients (Wan et al., 2018). Therefore, these results suggest that emotional factors may be involved in RD.

On the other hand, previous studies have found that additional electrical stimulation of the precuneus can cause dizziness in patients (Kahane et al., 2003; Wiest et al., 2004), which indicated that the precuneus may be the critical brain region for processing vestibular information, and the decreased functional activity of this brain region may lead to the decreased processing ability of vestibular information. Another study also reported that the precuneus was also involved in vision processing, closely related to the visuospatial circuit. This suggested that the precuneus was involved in multisensory interactions between the vestibule and the visual area (Della-Justina et al., 2015). Vision, vestibular sense, and proprioception was the three senses that maintain the balance of the human body. Problems with any of these senses would lead to an unstable balance of the human body (Van Nechel, 2017; Ponzo et al., 2018). Decreased function of the precuneus could cause the brain to process visual and vestibular information abnormally, resulting in an unstable balance in the body, which may be another reason for the appearance of RD symptoms. Fu et al. applied rs-fMRI to study the brain functional activities of patients with RD and also found that bilateral precuneus ALFF was significantly reduced in patients with RD (Fu et al., 2022). It further suggested that the precuneus dysregulation was due to the problems or maladaptation of visual-vestibular interaction in patients with RD. It may be an essential link causing the occurrence of RD symptoms.

### 4.2. The ALFF value increases in the right STG in patients with RD

The vestibular organ was a component of the inner ear and was crucial in transmitting sensory information related to head movements to the vestibular center (Duque-Parra, 2003). Due to limited knowledge of neuroanatomy at specific points in human history, the localization and function of the human vestibular cortex lacked concrete evidence (Duque-Parra, 2004). Recently, positron emission tomography (PET) and functional magnetic resonance imaging (fMRI) have been used to locate the vestibular cortex in the process of vestibular electrical stimulation, which has made up for the defect of human understanding of the vestibular cortex to some extent (Vitte et al., 1996; Lopez et al., 2012; Kirsch et al., 2019; Devantier et al., 2020). Research has discovered that, unlike other sensory cortices, the vestibular cortex consisted of a network of various brain regions (Dieterich and Brandt, 2018), including the parieto-insular vestibular cortex (PIVC), inferior parietal lobe (supramarginal gyrus and angular gyrus), temporal lobe (particularly the superior temporal gyrus), precuneus, frontal cortex, cingulate gyrus, hippocampus and other brain regions (Bucy, 1967; Lopez et al., 2012; zu Eulenburg et al., 2012; Dieterich and Brandt, 2015a,b; Frank and Greenlee, 2018; Nakul et al., 2021). Among these, the PIVC was considered the core region of the vestibular cortex (Dieterich and Brandt, 2008; Chen et al., 2010; Lopez and Blanke, 2011).

Our study used the ALFF analysis method to find enhanced brain functional activity in the right STG in RD patients. The STG was believed to be a part of the vestibular cortex that processes information from the peripheral vestibular information (Bucy, 1967; Nakul et al., 2021). Becker-Bense et al. found that vestibular syndrome caused by damage to the midbrain, thalamus, and vestibular cortex rarely presented as rotational vertigo, mainly as walking instability and balance disorder (Becker-Bense et al., 2016). Brandt et al. showed that the vestibular center had a particular compensatory potential. When the central vestibular function was impaired, the vestibular cortex and the corresponding areas under the cortex would carry out central compensatory action to eliminate the sense of vertigo (Brandt et al., 1997). The enhanced functional activity in the STG may be due to the continuous stimulation of the vestibular nerve on one side due to the flow of otolith particles in the semicircular canal in BPPV patients, and the vestibular function on both sides of the human body is always in an asymmetric state. The vestibular information was transmitted to the vestibular cortex through the vestibular nerve, vestibular nucleus, brain stem, thalamus, and other structures (Dieterich and Brandt, 2015a,b). The vestibular center will activate the compensatory mechanism and constantly adjust the working state to adapt to the changes in the peripheral vestibular function, resulting in enhanced functional activities in some areas of the vestibular cortex.

Correlation analysis revealed that the longer the duration of BPPV patients, the stronger the functional activities of the right superior temporal gyrus became, which, to a certain extent, indicates that the compensatory activities in the vestibular center will be enhanced with the prolongation of the duration of BPPV. However, there was a delay in the compensation function of the central nervous system, even when the otolith particles are fully returned to their normal position. The vestibule may take longer to compensate and adapt to the new equilibrium state (Faralli et al., 2016). Therefore, the enhancement of vestibular compensatory activity may be one of the causes of walking instability, floating sensation, neck tightness, and other symptoms in RD patients.

### 4.3. Changes in brain FC in patients with RD

Expanding on the ALFF analysis, our study investigated the FC of the brain in patients with RD using a seed-based FC method. It involved selecting one or multiple seed points, extracting the seed’s time series, and conducting correlation analysis with other voxels throughout the entire brain to obtain a comprehensive FC map (Joel et al., 2011; Smitha et al., 2017; Bonzano et al., 2022).

Our study utilized the right STG as the seed point for FC analysis. We identified a reduction in FC between the right STG, the right SMG, and the left precuneus. These regions, namely the STG, SMG, and precuneus, collectively form the vestibular cortex, which playde a vital role in processing and integrating peripheral vestibular information (Lopez et al., 2012). The human brain comprised various independent yet interconnected regions collaborating to carry out complex tasks (Park and Friston, 2013). Raiser et al. analyzed a comprehensive vestibular cortex brain network using structural and functional magnetic resonance imaging techniques. Their research unveiled a distinct modular organization within the structural partitioning of the vestibular cortex network, highlighting a high degree of connectivity within the hemisphere of the vestibular cortex. Decreased FC between these specialized areas could contribute to episodes of dizziness (Raiser et al., 2020). Our study observed diminished functional connectivity among brain regions within the vestibular cortex of patients with RD. This finding suggests that individuals with RD may exhibit abnormal integration of vestibular information within the central vestibular system to some extent. Acute unilateral vestibular central damage generally did not cause rotational vertigo, mainly manifested in Unsteady gait and balance disorders. Previous studies have believed that maintaining body balance required vestibular sense and the visual system. If the vestibular information was mismatched in the bilateral vestibular cortex, the perception of body position and movement is determined by the matching hemispheres of the vestibular and visual cortex (Dieterich and Brandt, 2015a,b). The FC between the right STG, the right SMG, and the left precuneus in patients with RD was reduced, which may reveals that the bilateral vestibular cortex of RD has a mismatch in processing vestibular information, but further studies are needed to confirm it.

### 4.4. Limitations of this study

There were some limitations in our study. Firstly, the sample size of our study was too small to represent the whole RD population. Secondly, we used a seed-based FC analysis method to explore the FC in the whole brain. We determined the seed points according to the ALFF results. However, there may be some deviation in the experimental results as the selection of seed points was different. Thirdly, all subjects in this study were collected from one hospital, which could not eliminate the bias caused by regional differences. Fourthly, no scale data was collected for HC in our study, so we did not know whether the scale scores of the HC are also correlated with the imaging data. Finally, our subjects were collected during the corona virus disease 2019 (COVID-19) pandemic, and social isolation and general anxiety may also be important factors contributing to psychological symptoms.

In future studies, we still need to expand the sample size, acquire clinical data of RD patients from different trial centers, and eliminate the influence of regional factors. In future studies, we will adopt more advanced functional magnetic resonance analysis methods to explore the brain functional activities of patients with RD. For example, the cortical analysis method will be used to explore the local functional activities or FC of the brain in RD patients to solve the problem that the volume-based Spatial analysis ignores the characteristics of the brain-stretching according to the cortex and improve the sensitivity and specificity of brain signal extraction. To compensate for the defects caused by the selection of seed points, the changes in FC between brain regions will be analyzed by constructing brain networks. To explore the topological properties of the brain network of RD patients, we will use the graph theory analysis method to more intuitively understand the working mode of the brain. Finally, we will conduct vestibular rehabilitation (VR) training or transcranial magnetic stimulation (TMS) therapy on enrolled RD patients and evaluate the therapeutic effect to provide more reference for future treatment of RD symptoms.

This study used rs-fMRI to study the brain functional activities of patients with RD and obtained specific positive results, which provided a deeper understanding of the disease from a functional perspective and had particular clinical value for diagnosing and treating RD. Firstly, this study confirmed that there were specific abnormalities in the brain functional activities of patients with RD, which broke the previous understanding that RD was only a peripheral disease and lays the theoretical foundation for treating RD in the future. Secondly, this study found a specific correlation between the course of disease in RD patients and the changes in brain functional activities. The longer the course of BPPV patients, the more significant the changes in brain functional activities. Therefore, this suggests that in future clinical diagnosis and treatment, we should treat patients with RD as soon as possible, using treatments such as vestibular rehabilitation training to speed up the compensatory activity of vestibular center in RD patients and avoid further deterioration of brain functional activities in RD patients, to alleviate the clinical symptoms of patients better. Finally, our study found that patients with RD had anxiety problems, and the higher the HAMA anxiety score, the weaker the functional activity of the brain function area (precuneus) integrating vestibular information in patients with RD, which suggests that in the treatment of RD, we should not only solve the dizziness symptoms of patients but also take into account the emotional problems of patients.

## 5. Conclusion

In conclusion, our study found that compared with HC, RD patients had abnormal local functional activities in the right precuneus and the right STG, and the FC between the right STG and the right SMG and the left precuneus was weakened. These changes may explain symptoms such as dizziness, discomfort in walking, floating sensation, and neck tightness in RD patients.

## Data availability statement

The original contributions presented in the study are included in the article/Supplementary material, further inquiries can be directed to the corresponding authors.

## Ethics statement

The studies involving humans were approved by The Second Affiliated Hospital Ethics Committee of Xuzhou Medical University. The studies were conducted in accordance with the local legislation and institutional requirements. The participants provided their written informed consent to participate in this study. Written informed consent was obtained from the individual(s) for the publication of any potentially identifiable images or data included in this article.

## Author contributions

CL: design trial, write paper, and f-MRI data processing. DL: investigate and acquire patient information. YL: guidance of statistical methodology. ZC: f-MRI data acquisition. XW: trial design guidance. HL: resource integration. KW: paper revision. TL: technical support. LX: data collation and analysis. LR: project management. All authors contributed to the article and approved the submitted version.

## Footnotes

^1^ http://rfmri.org/DPABI
